# Remote assessment of caries, MIH, and plaque on intraoral 3D scan images: Findings from the LIFE Child study

**DOI:** 10.1007/s00784-026-06939-z

**Published:** 2026-05-28

**Authors:** Nadja Stiller, Jana Schmidt, Christof Meigen, Anne Messerschmidt, Wieland Kiess, Mandy Vogel, Antje Körner, Ellen Schulz-Kornas, Rainer Haak

**Affiliations:** 1 Department of Cariology, Endodontology and Periodontology, University of Leipzig, Leipzig, Germany; 2 Medical Faculty, Center for Pediatric Research (CPL), Leipzig University, University Hospital for Children and Adolescents Leipzig, LIFE Child, Leipzig, Germany; 3 German Center for Child and Adolescent Health (DZKJ), partner site Leipzig/Dresden, Leipzig, Germany; 4 Helmholtz Institute for Metabolism, Obesity and Vascular Research (HI-MAG), Helmholtz Munich and University of Leipzig, Leipzig, Germany

**Keywords:** Dental Caries, Molar-Incisor Hypomineralisation, Child, Adolescent, Digital Dentistry, Intraoral Scanner

## Abstract

**Objectives:**

To evaluate the diagnostic agreement between clinical examinations and time-delayed remote diagnostic assessments of intraoral 3D scans (IOS) for detecting oral diseases in children and adolescents, validating intraoral 3D scans as a tool in epidemiology studies.

**Materials and methods:**

A total of 511 participants aged 7.5–20.5 years from the LIFE Child cohort received standardized chairside clinical diagnostics followed by intraoral 3D scanning (TRIOS 5, 3shape). Two calibrated examiners independently performed blinded remote review of digital scans after a predefined latency period. Diagnostic parameters comprised caries (ICDAS II), MIH classification, restorative status, sealant status, and plaque accumulation quantified using the Plaque Index (PI). Primary outcomes were diagnostic agreement and interrater reliability, analysed by weighted percent agreement, Cohen’s weighted Kappa and Bland-Altman plots.

**Results:**

Very high agreement was observed between clinical and IOS-based assessments for caries experience (DMF-T: $\:{pa}_{w}$= 0.990, κ = 0.77; dmf-t: $\:{pa}_{w}$= 0.991, κ = 0.93) and for severity-graded caries lesions (ICDAS 3–4: $\:{pa}_{w}$= 0.998, κ = 0.69; ICDAS 5–6: $\:{pa}_{w}$= 1.000, κ = 0.87). Agreement for initial lesions (ICDAS 1–2) was lower than for the more advanced lesions ($\:{pa}_{w}$= 0.979, κ = 0.29). Fissure sealants and fillings showed excellent agreement ($\:{pa}_{w}$= 0.997, κ = 0.84 and $\:{pa}_{w}$= 0.998, κ = 0.71). MIH detection on at least one tooth showed very good agreement ($\:{pa}_{w}$ = 0.932, κ = 0.83), with consistently high agreement across severity codes. Plaque assessment showed good agreement overall ($\:{pa}_{w}$ = 0.898), with slightly higher agreement in anterior regions. Interrater reliability was substantial to excellent across parameters.

**Conclusions:**

Intraoral 3D scans enable reliable remote assessment of clinically relevant dental conditions in children and adolescents, particularly for advanced caries lesions and molar incisor hypomineralisation (MIH).

**Clinical Relevance:**

Intraoral 3D scanning enables efficient remote evaluation in screening and epidemiological programs, prioritizing visually distinct or treatment-relevant findings, with slightly reduced alignment for early enamel findings.

## Introduction

Intraoral scanners (IOS) have become integral to digital workflows in modern dentistry, expanding from their initial applications in prosthodontics and orthodontics [1] into clinical screening and diagnostic domains [2]. IOS generate high-fidelity three-dimensional surface models, support digital treatment planning, shade mapping, and the creation of fixed dental restorations, and enable morphological hard and soft tissue assessment [2]. Recent studies have identified IOS as a viable alternative to traditional visual-tactile examination for the detection of carious lesions, non-carious tooth structure loss, and the assessment of oral hygiene [3–5]. Moreover, IOS allow reliable visualization of plaque accumulation, supporting monitoring of oral hygiene [5–9]. Their integration into large-scale epidemiological studies, such as the LIFE-Child study [10, 11], may reduce examination time and personnel costs.

Visual-tactile examination performed chairside by dental professionals remains the reference standard for clinical diagnosis [12–14], but requires time, on-site deployment, and the physical availability of a dentist [15]. In contrast, remote diagnostic review based on IOS enables blinded, time-delayed evaluations by dentists and eliminates the need for the simultaneous presence of the patient and the examiner [16]. The diagnostic accuracy of IOS is particularly high in caries detection and can potentially be enhanced by adjunct technologies such as fluorescence imaging [17, 18] or transillumination [19] by facilitating the differentiation between healthy and carious tissues. Comparative studies have further shown that intraoral scanners can visualize enamel demineralization and proximal lesions and may therefore represent a diagnostic alternative to bitewing radiography [20, 21]. Furthermore, the scans can be stored and compared longitudinally, supporting the visualization and monitoring of lesion development over time during follow-up examinations. This capability is particularly relevant in pediatric dentistry, where IOS-based examinations provide a non-invasive and radiation-free diagnostic approach and are generally well accepted by younger patients [22].

While surface-based quantification of 3D dental models has shown to be reliable in detecting and assessing carious lesions and erosions, the diagnosis of enamel defects in molar-incisor hypomineralization (MIH) remains underexplored. To date, only a single study by Schulz-Weidner et al. (2025) [23] has examined MIH using IOS. In that study, MIH was recorded exclusively as a dichotomous outcome, without any clinical subclassification, severity grading, or morphological characterization of the defects.

The aim of this study was to evaluate the diagnostic agreement between clinical examinations and time-delayed remote diagnostic assessments of intraoral 3D scans (IOS) for detecting oral diseases in children and adolescents, validating intraoral 3D scans as a tool in epidemiology studies. A secondary aim was to explore whether agreement varied according to disease severity.

## Materials and methods

A total of 511 participants aged 7.5 to 20.5 years from the LIFE Child study were included in this investigation, which was conducted between June and December 2023. The population-based cohort study LIFE Child has been described in detail previously [10, 11]. The study followed the Declaration of Helsinki and was approved by the Ethics Committee of the University of Leipzig (Reg. No. 477/190-ek).

**Fig. 1:**
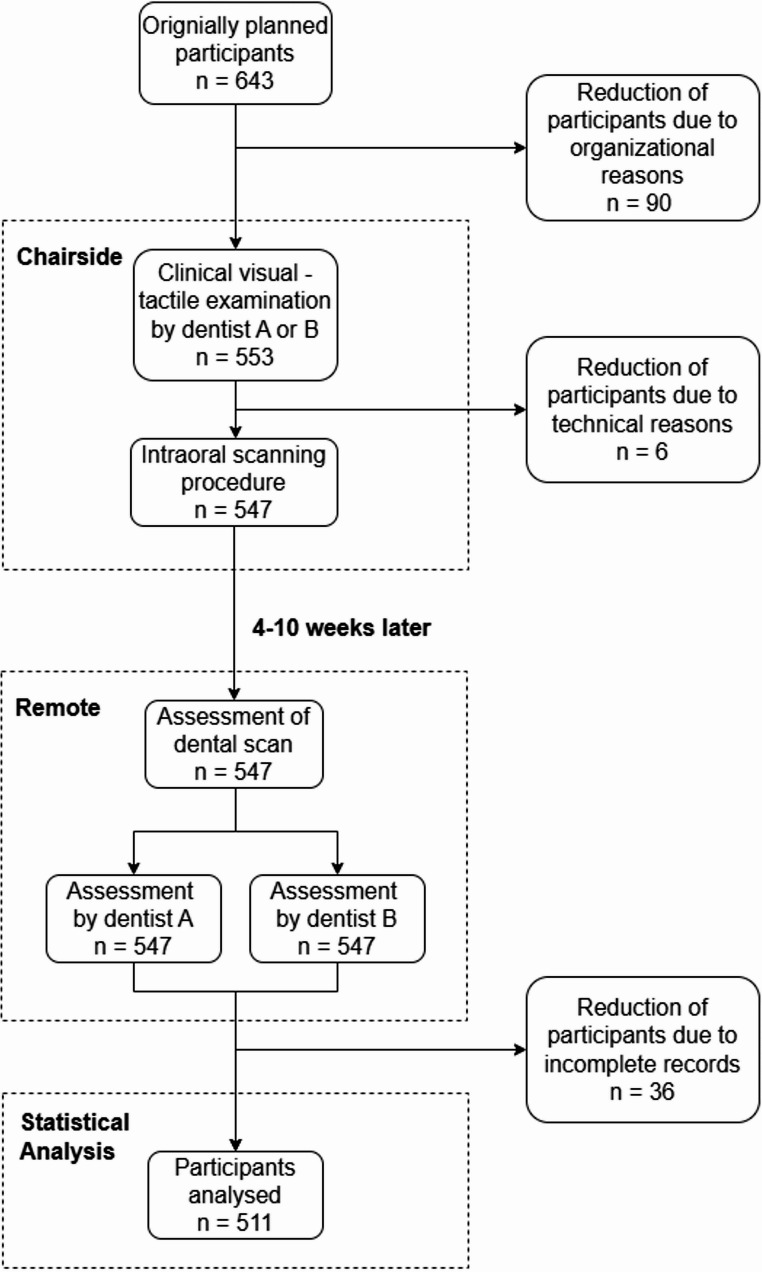
Flowchart which illustrates recruitment of participants and examination procedures, chairside and remote

Clinical dental examiners were trained at study onset, and their assessments were calibrated. Inter-examiner reliability was excellent ($\:{pa}_{w}$= > 0.980; κ > 0.88), as was intra-examiner reliability ($\:{pa}_{w}$= > 0.969; κ > 0.87). Both examiners were educated at the same university and completed their education and examinations concurrently. Examiners A and B were involved in the study, which consisted of two parts: a clinical visual-tactile examination of the participants and a visual examination based on dental scans (Fig. 1).

The examinations were carried out on a dental chair (Denta-Chair 303, BPR Swiss) with optimal lighting provided by a head lamp (Petzl Actic Core, 600 lm). The clinical inspection was performed using a probe, an oral mirror, and a WHO periodontal probe with a ball tip. Saliva aspirators and a dental 3-way air-water syringe (Pro Vac, BPR Swiss) were used to dry the teeth. There was no use of magnifying glasses. The intraoral scans were acquired using the TRIOS 5 Move scanner (3shape, Copenhagen, Denmark).

### Intraoral scanning

A 3D dental scan was taken with the TRIOS 5 from 3shape after the participants had habitually brushed their teeth. The room was darkened, and the lights were turned off. The participants were seated upright on the treatment chair. After the surfaces of the teeth were dried, the lower jaw was scanned first, followed by the upper jaw. The bite was scanned right and then left. The manufacturer’s recommended scanning strategy has always been followed. The scanning process was completed when the entire tooth status had been imaged. Care was taken to limit the number of images per scan to a maximum of 1500, in accordance with previous studies indicating that higher image counts and rescanning may affect processing performance and accuracy [24, 25].

### Assessment of oral hygiene

Oral hygiene was assessed using the Silness and Löe plaque index (PI) [26] on index teeth 16, 11, 26, 36, 31, 46 at the mesiobuccal, centrobuccal, distobuccal, palatal, and lingual sites. In principle, the choice of index teeth followed the Ramfjord teeth criteria [27]. In each quadrant, the first molars were considered because of the participants’ ages and because premolars had not yet erupted in some cases.

Plaque levels were assessed without staining using the following grading system: 0 (no plaque), 1 (thin film of plaque along the gingival margin, detectable by probing), 2 (moderate plaque along the gingival margin, visible, with clear interdental spaces) and 3 (abundant plaque along the gingival margin, with interdental spaces filled with plaque) [26]. For each participant, the PI was calculated as the mean of all site-specific scores, yielding a possible range from 0 to 3, with higher values indicating poorer oral hygiene. Separate PI scores were additionally derived for the anterior and posterior regions.

### Dental caries assessment

All surfaces of permanent teeth (occlusal/incisal, oral, buccal, mesial, distal) were assessed by visual inspection according to the ICDAS II (“International Caries Detection and Assessment System”, ICDAS 2005) codes [28]. The examined sites were diagnosed as follows: 0 = sound, 2 = distinct visual change in enamel (Codes 1 and 2 were combined and classified as initial caries lesions), 3 = localized enamel breakdown, 4 = non-cavitated surface with dentin shadow, 5 = distinct cavity with visible dentin, 6 = extensive cavity with visible dentin. In addition, fissure sealants and fillings were included in the study. For each participant, the total number of sites corresponding to each diagnostic code was calculated. Primary teeth and their surfaces were labelled as “carious” or “filled” without differentiation based on ICDAS codes.

The DMF/dmf index at tooth level was used to assess caries experience for both permanent and primary teeth. A tooth was classified as a DMF/dmf tooth if any of its surfaces showed caries or fillings (D/d component: decayed due to caries (≥ ICDAS 3), M/m: missing due to carious destruction, F/f: filled surface). In accordance with WHO recommendations, caries diagnosis was based on visual examination only [29].

### Evaluation of molar-incisor-hypomineralisation

Molar incisor hypomineralisation was assessed in permanent teeth. The severity was classified into four levels: 1 = opacity (> 2 mm), 2 = enamel disintegration, 3 = atypical restoration, and 4 = extraction due to MIH [30].

All permanent teeth were included, and the number of teeth assigned to each respective code was summed. In addition, the most severe findings were documented separately for teeth 16, 12, 11, 21, 22, 26, 36, 32, 31, 41, 42, and 46.

### Visual assessment of the dental scan

Four to ten weeks after the clinical examination, the same examiners assessed oral health using 3D scans on the laptop in the same examination room under natural lighting. A consistent screen brightness of 100% was maintained on the laptop. The 3D scans were adjusted, zoomed, and rotated to ensure optimal viewing conditions. Each scan was analysed in approximately 10–15 min. To reduce potential fatigue, the number of scans analysed per day was limited to 20. Dentists A and B analysed each dental scan individually to facilitate intra- and interpersonal comparisons with the clinical examination (Fig. 1).

The examination of the dental scans followed the same procedure as the clinical examination.

### Statistical analysis

Statistical analyses were performed using R (version 4.3.1) [31]. The following packages were used: dplyr, tidyr, ggplot2, psych, irr, irrCAC, purr and BlandAltmanLeh.

Descriptive statistics (mean, standard deviation, absolute and relative frequencies) were calculated for clinical and scan-based findings by age group (7.5–11.9 years, 12.0–15.9 years, and ≥ 16 years) and sex. Agreement between clinical assessments and IOS-based evaluations, as well as interrater agreement between the two examiners, was quantified using the weighted percent agreement $\:{pa}_{w}$ [32] and linearly Cohen’s weighted Kappa *κ* [33]. All variables were coded according to predefined maximum category levels. A Bland–Altman analysis with 95% limits of agreement (± 1.96 SD) was used for selected variables [34]. Figures were generated using ggplot2 [35].

We used weighted percent agreement rather than Cohen’s weighted Kappa because the latter adjusts for chance based on an assumption of statistical independence between raters, which is inconsistent with the context of trained dentists from the same school and the same teeth as a base. Further, Kappa is strongly influenced by marginal distributions, potentially yielding misleading results when prevalence is uneven, which is the case, as we found many more lower ratings than higher ones. Therefore, weighted percent agreement provides a more direct and interpretable measure of reliability for our ordinal data that accounts for the degree of (dis)agreement without imposing an invalid independence assumption [36].

For the calculation of weighted percent agreement, maximum possible scores (denominators) were predefined for each variable according to the number of teeth and surfaces examined and the respective scoring systems (Table 2).

For caries experience in primary teeth (dmft and its components d-t, m-t, f-t), a maximum score of 12 was applied. This reflects the study population, which included children in the resting mixed dentition phase, the second mixed dentition phase and the permanent dentition.

For DMFT and its components (D-T, F-T) the maximum score was set to 28, corresponding to the maximum number of permanent teeth examined (excluding third molars).

For all surface-based variables (including ICDAS categories, sound surfaces, fissure sealants, and restorations), a maximum score of 140 was used, based on the assessment of up to 28 teeth with 5 surfaces per tooth. The use of a fixed denominator of 12, 28 and 140 for all participants is a simplification, the potential overestimation it introduces is minimal given the low prevalence of affected teeth and surfaces and is not expected to alter the interpretation of the findings.

The variable “MIH on at least one tooth” was analysed as a binary outcome and therefore had a maximum score of 1 (yes = 1). For the individual MIH severity codes (Codes 1–3), a maximum score of 28 was applied, as all permanent teeth were evaluated. For analyses at the level of individual index teeth (16, 11, 21, 26, 36, 31, 41, 46), the maximum score was 4, corresponding to the highest severity level defined by the EAPD MIH classification.

The Plaque Index (PI) was initially calculated as an average across multiple tooth surfaces and teeth, yielding continuous decimal values. However, because the calculation of weighted percent agreement requires discrete, ordinal-scale data, the PI scores were converted into equivalent integer sum scores by multiplying them by the number of underlying surface assessments (total, anterior, and posterior) and rounding to whole numbers. These transformed scores correspond to summed ordinal surface-level ratings and are represented on discrete scales with defined theoretical ranges: total PI (0–72), anterior PI (0–24), and posterior PI (0–48). This transformation was performed exclusively for the weighted percent agreement analysis and does not alter the clinical interpretation of the PI scores.

## Results

### Descriptive clinical findings

A total of 511 participants were included, with an approximately balanced sex distribution across age groups. The mean DMF-T was 0.24 (SD 0.81) in participants aged 7.5–11.9 years, 0.84 (SD 1.53) in those aged 12–15.9 years, and 1.99 (SD 2.47) in participants aged ≥ 16 years. The proportion of participants with molar–incisor hypomineralization (MIH) was 26.36%, 27.96% and 20.95% in the respective age groups. The mean total plaque index (Silness and Löe) was 0.88 (SD 0.43), 0.77 (SD 0.43), and 0.62 (SD 0.44; Table 1).

### Comparison of clinical and scanner-based findings

Clinical and remote (IOS image-based) examinations demonstrated very good agreement for all diagnostic parameters. The weighted percent agreement ($\:{pa}_{w})$ for the DMF-T index was $\:{pa}_{w}$ = 0.990, and $\:{pa}_{w}$ = 0.991 for the dmf-t index (Table 2). The separate components showed very good to excellent agreement (D-T: $\:{pa}_{w}$ = 0.993, F-T: $\:{pa}_{w}$ = 0.996, d-t: $\:{pa}_{w}$ = 0.994, m-t: $\:{pa}_{w}$ = 0.999, f-t: $\:{pa}_{w}$ 0.994; Table 2).

**Fig. 2:**
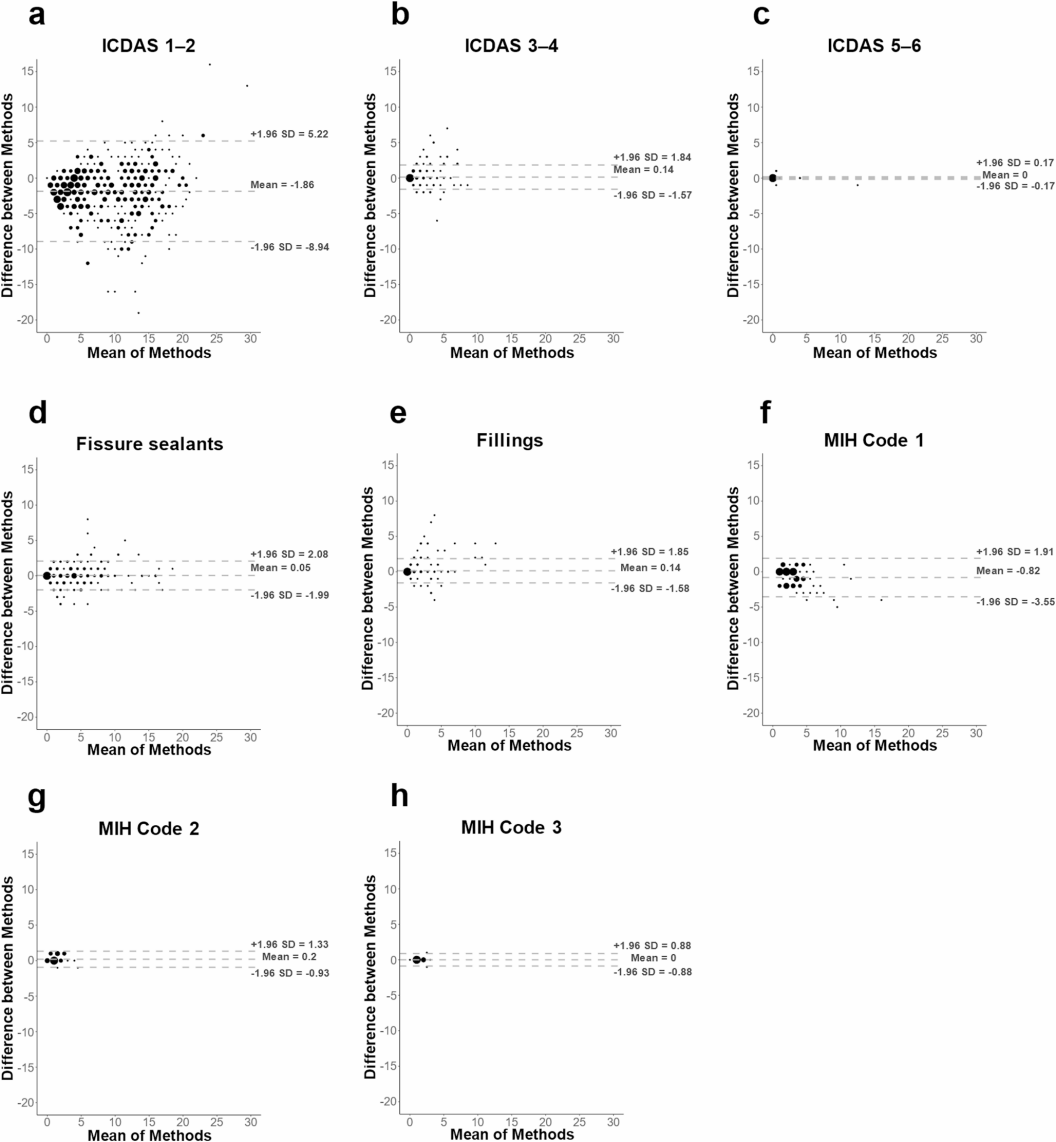
Bland-Altman plots comparing clinical examination (method 1) and scan-based examination (method 2) performed by a single rater, **a–e**: surface-based detection of caries lesions (ICDAS 1–6), fissure sealants and fillings on permanent teeth, **f-h**: tooth-based detection of MIH (Code 1–4) on permanent teeth, Code 1: Opacity, Code 2: Enamel disintegration, Code 3: Atypical restoration

Considering the ICDAS assessment at the surface level, the proportion of sound surfaces (ICDAS 0) differed by no more than 3% between the examination methods and showed substantial agreement ($\:{pa}_{w}$ = 0.978). Extensive lesions (ICDAS 5–6) were detected with complete agreement ($\:{pa}_{w}$ = 1.000), with the corresponding Bland-Altman plot showing tight clustering around the mean and no systematic bias (Fig. 2c).

Moderate lesions (ICDAS 3–4) yielded very high agreement ($\:{pa}_{w}$ = 0.998) but slightly decreased κ = 0.69, also reflected in increased variability and isolated outliers in the Bland-Altman plot (Fig. 2b). Agreement was lower for initial lesions (ICDAS 1–2) with $\:{pa}_{w}$ = 0.979 and κ = 0.29 (Table 2). Also, the Bland-Altman plot showed greater dispersion of differences between the two methods (Fig. 2a).

Fissure sealants and fillings were detected with excellent consistency, with $\:{pa}_{w}$ = 0.997 and $\:{pa}_{w}$ = 0.998. Corresponding Bland-Altman plots (Fig. 2d and e) supported these findings, with narrow limits of agreement and minimal deviations.

The prevalence of MIH was higher in the IOS-based assessments compared to clinical examination. Opacities (Code 1) were the most common manifestation in both methods, but were detected more frequently in the IOS-based assessments (e.g., 192 teeth [3.93%] vs. 87 teeth [1.78%] in participants aged 12–15.9 years). Severe forms such as enamel breakdown (Code 2) and atypical restorations (Code 3) were also more frequently recorded with IOS (Table 1).

Overall, MIH diagnosis on at least one tooth showed very good agreement between clinical and IOS assessments ($\:{pa}_{w}$ = 0.932). Agreement varied across MIH severity codes, with $\:{pa}_{w}$ = 0.961 for opacities (Code 1), and higher values for enamel breakdown (Code 2) $\:{pa}_{w}$ = 0.987 and atypical restorations (Code 3, $\:{pa}_{w}$ = 0.994).

For MIH-related extractions (Code 4), the number of observations was too small (*n* = 3) to permit a valid reliability analysis.

Tooth-level analysis for MIH yielded weighted percent agreement values ranging from $\:{pa}_{w}$ = 0.932 (tooth 41) to $\:{pa}_{w}$ = 0.982 (tooth 36, Table 2).

Plaque assessment using the Silness & Löe Plaque Index [26] showed good agreement between clinical and scan-based methods ($\:{pa}_{w}$ = 0.898). Agreement was higher in anterior teeth ($\:{pa}_{w}$ = 0.887) compared to posterior regions ($\:{pa}_{w}$ = 0.881; Table 2).

### Interrater reliability in IOS-based examination

The evaluation of 3D intraoral scans by two independent examiners (dentist A and dentist B) demonstrated overall high interrater reliability. For the DMF-T index, a weighted percent agreement of $\:{pa}_{w}$ = 0.990 was achieved; while in primary dentition, the dmf-t index showed $\:{pa}_{w}$ = 0.985 (Table 2). Agreement was complete for extensive lesions (ICDAS 5–6, $\:{pa}_{w}$ = 1.000), and lower for initial lesions (ICDAS 1–2; $\:{pa}_{w}$ = 0.980). Very good agreement was also observed for fissure sealants ($\:{pa}_{w}$ = 0.997) and fillings ($\:{pa}_{w}$ = 0.998). Interrater reliability for MIH diagnosis on at least one tooth was generally good ($\:{pa}_{w}$ = 0.900), with the highest agreement recorded for atypical restorations (Code 3: $\:{pa}_{w}$= 0.996).

**Table 1:** Descriptive characterisation of the study cohort, percentages for participant characteristics refer to the proportion within each age group. For ICDAS and MIH findings, percentages refer to the proportion of affected tooth surfaces within the respective age group

	Age 7.5–11.9		Age 12-15.9		Age 16+	
*n* total	220		186		105	
Sex, n (%)	Males, 119 (54.1) Females, 101 (45.9)		Males, 91 (48.9) Females, 95 (51.1)		Males, 53 (50.5) Females, 52 (49.5)	
	**clinical**	**scan**	**clinical**	**scan**	**clinical**	**scan**
Permanent teeth overall, n	**3089**	**3085**	**4889**	**4883**	**2894**	**2893**
Permanent teeth per patient, mean (SD)	14.04 (4.56)	14.02 (4.57)	26.28 (3.42)	26.25 (3.41)	27.56 (1.16)	27.55 (1.07)
Primary teeth overall, n	2071	2056	144	143	8	8
Primary teeth per patient, mean (SD)	9.41 (4.23)	9.35 (4.19)	0.77 (2.16)	0.77 (2.17)	0.08 (0.38)	0.08 (0.38)
DMF-T, mean (SD)	0.24 (0.81)	0.20 (0.68)	0.84 (1.53)	0.71 (1.56)	1.99 (2.47)	1.79 (2.23)
D-T, mean (SD)	0.20 (0.76)	0.16 (0.63)	0.50 (1.11)	0.42 (1.14)	1.16 (1.66)	0.99 (1.53)
M-T, mean (SD)	0.00 (0.00)	0.00 (0.00)	0.01 (0.07)	0.01 (0.07)	0.01 (0.10)	0.01 (0.10)
F-T, mean (SD)	0.04 (0.22)	0.03 (0.20)	0.34 (0.95)	0.28 (0.80)	0.82 (1.65)	0.82 (1.55)
dmf-t, mean (SD	1.00 (1.83)	0.97 (1.86)	0.39 (0.68)	0.47 (0.88)	0.40 (0.55)	0.40 (0.55)
ICDAS (surface-related, permanent teeth) All surfaces, n	**15,445**	**15,425**	**24,445**	**24,415**	**14,470**	**14,465**
Sound (ICDAS 0), n (%)	14,593 (94.48)	14,213 (92.14)	22,349 (91.43)	21,941 (89.87)	13,002 (89.85)	12,854 (88.86)
Initial caries (ICDAS 1–2), n (%)	802 (5.19)	1171 (7.59)	1966 (8.04)	2369 (9.70)	1316 (9.09)	1494 (10.33)
Moderate caries (ICDAS 3–4), n(%)	46 (0.30)	37 (0.24)	114 (0.47)	88 (0.36)	147 (1.02)	113 (0.78)
ICDAS 3, n (%)	39 (0.25)	32 (0.21)	88 (0.36)	75 (0.31)	112 (0.77)	98 (0.68)
ICDAS 4, n (%)	7 (0.05)	5 (0.03)	26 (0.11)	13 (0.05)	35 (0.24)	15 (0.10)
Extensive caries (ICDAS 5–6), n(%)	4 (0.03)	4 (0.03)	16 (0.07)	17 (0.07)	5 (0.03)	4 (0.03)
ICDAS 5, n (%)	4 (0.03)	4 (0.03)	5 (0.02)	5 (0.02)	5 (0.03)	4 (0.03)
ICDAS 6, n (%)	0 (0.00)	0 (0.00)	11 (0.04)	12 (0.05)	0 (0.00)	0 (0.00)
Fissure sealants, n (%)	377 (2.44)	392 (2.54)	656 (2.68)	639 (2.62)	488 (3.37)	466 (3.22)
Fillings, n (%)	26 (0.17)	17 (0.11)	118 (0.48)	81 (0.33)	146 (1.01)	123 (0.85)
Participants with MIH, n (%)	58 (26.36)	67 (30.45)	52 (27.96)	54 (29.03)	22 (20.95)	28 (26.67)
Number of teeth with MIH Code 1: opacity, n (%)	104 (3.37)	199 (6.45)	87 (1.78)	192 (3.93)	50 (1.73)	123 (4.25)
Code 2: enamel disintegration, n (%)	20 (0.65)	26 (0.84)	15 (0.31)	25 (0.51)	5 (0.17)	12 (0.41)
Code 3: atypical restoration, n (%)	7 (0.23)	10 (0.32)	8 (0.16)	13 (0.27)	3 (0.10)	6 (0.21)
Code 4: extracted due to MIH, n (%)	0 (0.00)	0 (0.00)	2 (0.04)	1 (0.02)	0 (0.00)	0 (0.00)
PI Score, Silness & Löe 1964 total, mean (SD)	0.88 (0.43)	0.77 (0.23)	0.77 (0.43)	0.73 (0.24)	0.62 (0.44)	0.70 (0.22)
anterior teeth, mean (SD)	0.70 (0.57)	0.47 (0.31)	0.61 (0.49)	0.46 (0.31)	0.52 (0.48)	0.45 (0.24)
posterior teeth, mean (SD)	0.97 (0.45)	0.93 (0.25)	0.85 (0.48)	0.87 (0.26)	0.67 (0.48)	0.83 (0.26)

**Table 2:** Weighted percent agreement ($\:{pa}_{w}$; proportion scale 0–1, where 1.0 = 100% agreement) and Cohen’s weighted Kappa *κ* between clinical and intraoral-scan-based assessment for the different outcome parameters

Variable	Denominator for calculation of $\:{\boldsymbol{p}\boldsymbol{a}}_{\boldsymbol{w}}$ (*n*)	$\:{\boldsymbol{p}\boldsymbol{a}}_{\boldsymbol{w}}$ clin vs. scan (95% CI)	$\:{\boldsymbol{p}\boldsymbol{a}}_{\boldsymbol{w}}$ scan vs. scan (95% CI)	κ clin vs. scan	κ scan vs. scan
Primary teeth dmf-t	12	0.991, (0.987,0.995)	0.985, (0.979,0.991)	0.93	0.89
d-t	12	0.994, (0.991,0.998)	0.990, (0.985,0.994)	0.88	0.76
m-t	12	0.999, (0.998,1)	0.998, (0.997,1)	0.95	0.91
f-t	12	0.994, (0.991,0.998)	0.991, (0.987,0.995)	0.92	0.88
Permanent teeth DMF-T	28	0.990, (0.987,0.992)	0.990, (0.988,0.992)	0.77	0.77
D-T	28	0.993, (0.991,0.994)	0.993, (0.991,0.994)	0.72	0.73
F-T	28	0.996, (0.994,0.997)	0.996, (0.995,0.997)	0.77	0.74
Sound (ICDAS 0)	140	0.978 (0.976,0.98)	0.978, (0.976,0.98)	0.86	0.86
Initial caries (ICDAS 1–2)	140	0.979, (0.977,0.981)	0.980, (0.979,0.982)	0.29	0.42
Moderate caries (ICDAS 3–4)	140	0.998, (0.997,0.998)	0.998, (0.998,0.999)	0.69	0.74
ICDAS 3	140	0.998, (0.998,0.999)	0.999, (0.998,0.999)	0.66	0.73
ICDAS 4	140	0.999, (0.999,1)	0.999, (0.999,1)	0.48	0.34
Extensive caries (ICDAS 5–6)	140	1.000, (1,1)	1.000, (1,1)	0.87	0.80
ICDAS 5	140	1.000, (1,1)	1.000, (1,1)	0.84	0.73
ICDAS 6	140	1.000, (1,1)	1.000, (1,1)	0.67	0.67
Fissure sealants	140	0.997, (0.996,0.997)	0.997, (0.996,0.997)	0.84	0.86
Fillings	140	0.998, (0.998,0.999)	0.998, (0.998,0.999)	0.71	0.66
At least one tooth with MIH	1	0.932, (0.91,0.953)	0.900, (0.874,0.926)	0.83	0.76
Number of teeth with MIH Code 1: opacity	28	0.961, (0.952,0.971)	0.942, (0.931,0.953)	0.55	0.61
Code 2: enamel disintegration	28	0.987, (0.98,0.994)	0.985, (0.976,0.994)	0.60	0.69
Code 3: atypical restoration	28	0.994, (0.984,1)	0.996, (0.99,1)	0.67	0.81
Tooth 16	4	0.973, (0.95,0.996)	0.971, (0.951,0.99)	0.65	0.78
Tooth 11	4	0.971, (0.929,1)	0.977, (0.945,1)	0.67	0.73
Tooth 21	4	0.974, (0.937,1)	0.985, (0.955,1)	0.59	0.62
Tooth 26	4	0.942, (0.896,0.989)	0.967, (0.945,0.989)	0.56	0.71
Tooth 36	4	0.982, (0.961,1)	0.984, (0.968,0.999)	0.65	0.78
Tooth 31	4	0.946, (0.887,1)	0.964, (0.914,1)	0.51	0.67
Tooth 41	4	0.932, (0.857,1)	0.964, (0.883,1)	0.39	0.55
Tooth 46	4	0.981, (0.96,1)	0.978, (0.959,0.997)	0.61	0.75
PI Score total	72	0.898, (0.892,0.905)	0.936, (0.932,0.941)		
anterior	24	0.887, (0.878,0.896)	0.922, (0.917,0.928)		
posterior	48	0.881, (0.873,0.889)	0.928, (0.923,0.933)		

**Fig. 3:**
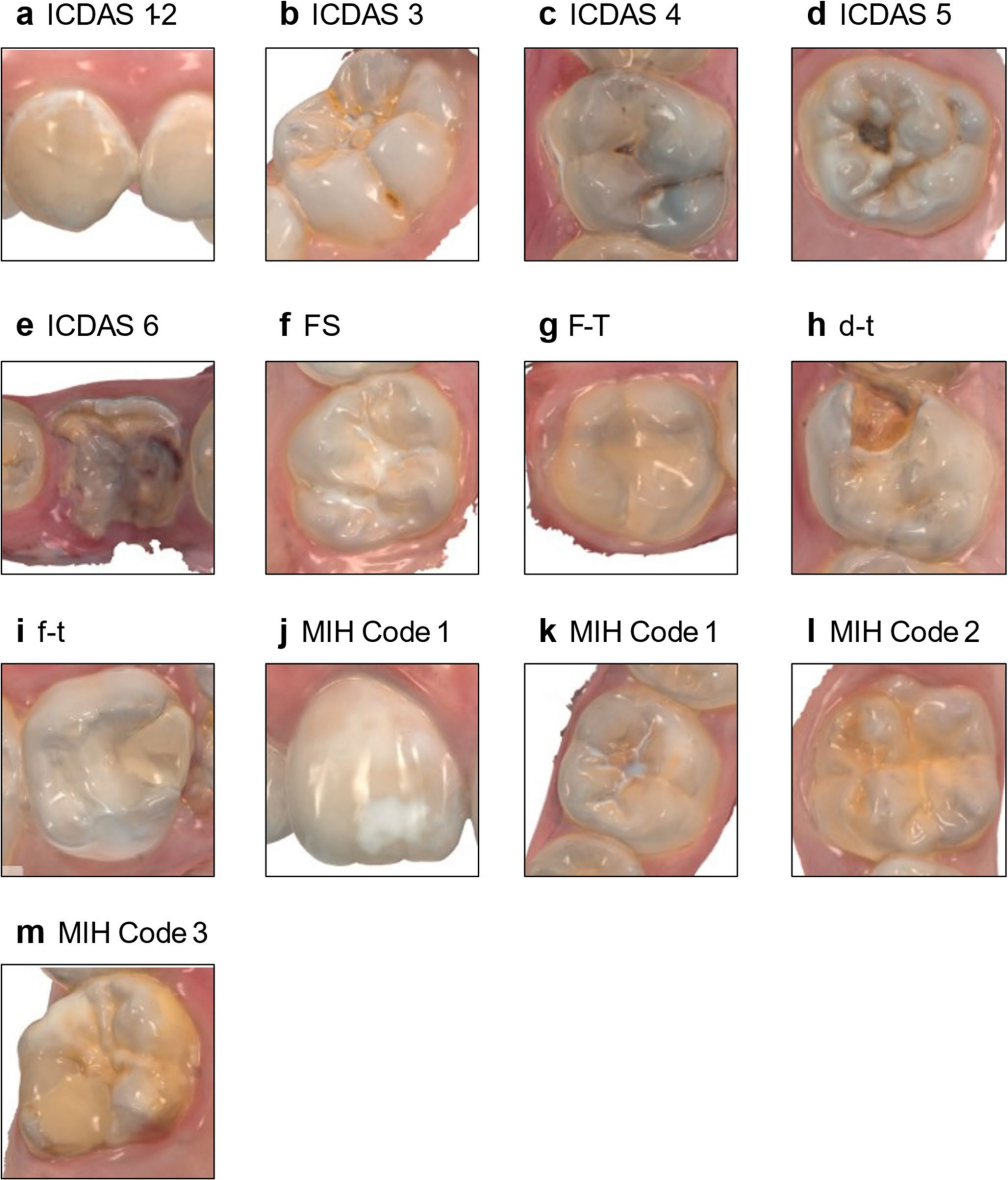
Representative IOS images of the dental findings considered in the present study. FS= fissure sealant, F-T = filled permanent tooth, d-t = decayed primary tooth, f-t = filled primary tooth

## Discussion

The primary objective of the present study was to systematically compare visual-tactile findings of chairside examinations with those obtained from blinded, time-delayed remote assessment of intraoral 3-D scans. In the present study, the diagnostic potential of an IOS was evaluated solely from three-dimensional surface images. By focusing on IOS data, we aimed to assess the clinical interpretability of anatomical details without further enhancement, filtering or postprocessing. Inspired by the study of Alaraudanjoki et al. (2017) [16] who validated erosive wear assessments on 3D models using the BEWE index we systematically compared diagnostic agreement between chairside clinical examination and blinded, time-delayed digital review of intraoral scans in a population-based pediatric and adolescent cohort.

Very good agreement was found between clinical and IOS-based assessments for caries experience in the primary and permanent dentition. IOS-based reliability patterns observed here are consistent with diagnostic agreement for the TRIOS 4 scanner reported by Schulz-Weidner et al. (2024) [22] (dmf-t: κ = 0.714; DMF-T: κ = 0.680), and with their more recent teledentistry study from 2025 [23], which demonstrated almost perfect agreement between visual and IOS-based caries assessments (κ = 0.965–0.995) in children. Comparable levels of agreement have also been reported in older adults [38]. Strong diagnostic alignment was also evident across ICDAS categories, particularly for severity-graded lesions, highlighting the scanner’s ability to differentiate severely affected surfaces clearly. Evidence from Metzger et al. (2022) further confirmed high concordance of cavitated proximal carious lesions on near-infrared imaging and bitewing radiographs [21], which aligns with the high agreement observed for cavitated and advanced lesion stages.

Clinically, moderate-to-extensive lesions often require preventive or restorative care. The present findings indicate that intraoral 3D surface scans can serve as a scalable tool for remote, examiner-independent remote screening, particularly in school settings and large epidemiological studies where dental resources, particularly dentists, are limited and advanced hard-tissue findings are prioritized.

The diagnostic performance of remote rating for early lesions (ICDAS 1–2, Fig. 3a) was the weakest across the ICDAS categories, a distribution consistent with findings from Michou et al. (2021) [18] and Sá et al. (2024) [39]. The poor visibility of initial lesions is probably related to the low optical contrast with sound enamel. The diagnostic challenge of detecting early caries lesions is also found in clinical assessment [40, 41]. Interestingly, the lower diagnostic agreement for early-stage caries reported in the literature was not fully reflected in our data. Despite the broader dispersion in the Bland–Altman analysis (Fig. 2a), the weighted percent agreement for ICDAS 1–2 remained high. The corresponding kappa value was low, which is expected given the very low prevalence of initial lesions in the sample and the known of kappa to skewed category distributions [37].

Although our findings suggest that subtle enamel changes can already be reliably identified on IOS-based surface scans, further improvement of early-stage detection remains possible. Studies have shown that augmenting IOS systems with fluorescence [17], transillumination [19], or near-infrared imaging [42] significantly improves the sensitivity for detecting initial caries lesions. Additionally, emerging AI-based interpretation tools have shown promise in increasing diagnostic consistency, particularly for subtle enamel changes [15, 43].

### MIH detection

MIH prevalence in the cohort reached 25.8%, exceeding the DMS-6 estimates (15.3%) [44], while remaining within internationally documented prevalence ranges [45, 46].

The present findings show that MIH prevalence was consistently higher in scan-based assessments compared to clinical examinations, particularly for opacities (Code 1), as shown in Fig. 3j (molar) and Fig. 3k (incisor). Intraoral 3D scans may offer improved sensitivity for detecting subtle enamel defects, possibly due to the ability to manipulate digital models (zoom, rotate) and re-examine areas repeatedly under optimal viewing conditions. Such advantages may increase detection rates, especially for minor defects that might be missed in a time-constrained clinical setting.

The increased detection must be interpreted with caution, as no structural reference standard was available for MIH verification. Surface reflections, color rendering, and varying lighting conditions may increase classification uncertainty in mild Code 1 lesions. Future studies should address these limitations by using additional methods to confirm hypomineralization at the structural level.

The overall agreement between clinical and scan-based MIH assessments was very good, considering the detection of the presence of MIH on at least one tooth supporting the feasibility of IOS for severity-graded documentation and monitoring of MIH lesions. Comparable findings were reported by Schulz-Weidner et al. (2025) [23], who demonstrated almost perfect agreement for MIH detection based on IOS in a pediatric population (κ = 0.996).

Agreement between the methods was high across all MIH severity grades and tended to be slightly higher for more severe MIH manifestations (Table 2). These results confirm our hypothesis that the more pronounced and morphologically distinct the MIH lesion is, the higher the diagnostic consistency between the two methods. These results align with recent findings by Neumayr et al. (2024) [47], who showed that deep learning-based processing of intraoral 2D images can enhance the detection of MIH-related enamel defects.

Overall, IOS-based assessments showed high interrater reliability across MIH codes, including early stages. Although no objective reference standard was applied, the intra- and inter-method concordance provides evidence of construct validity for severity-graded MIH assessment. As MIH gains public health relevance, IOS documentation could provide a valuable tool for early detection, patient communication, and long-term monitoring in pediatric care.

### Detection of restorations and sealants

Fissure sealants showed excellent agreement. They are typically applied to smooth, easily accessible occlusal surfaces, exhibit a distinct optical appearance and homogeneity, making them readily identifiable in both clinical and scan-based assessments (Fig. 3f). This is in line with the findings of Xiong et al. (2024) [48] who showed that deep learning algorithms can reliably and accurately detect fissure sealants in intraoral photographs.

Restorations likewise showed very high agreement between clinical and IOS-based assessments. This finding is consistent with the results of Schulz-Weidner et al. (2025) [23], who also reported almost perfect IOS-based detection of restorations in children (κ = 0.988–0.993). Bland–Altman limits were slightly wider than for sealants (Fig. 2d), as composite restorations often exhibit minimal contrast to the adjacent enamel (Fig. 2e), making their identification more challenging [49].

The differentiation between fissure sealants and small composite restorations, particularly in first permanent molars, can be challenging in both clinical and digital assessments. In minimally invasive or atraumatic restorative approaches, the optical distinction may be subtle. Because the tactile component at restoration margins is absent in remote diagnostics, accurate identification can be further complicated. Although confirmation via dental records was not available in the remote assessment, the high agreement observed suggests that misclassification was limited. Nonetheless, this represents a potential source of diagnostic uncertainty.

### Plaque assessment and oral hygiene monitoring

Plaque accumulation declines with age [50, 51], consistent with longitudinal behavioral trends observed with advancing adolescence. Agreement between clinical and IOS-based plaque assessments was good. The slightly higher agreement in the anterior region is in line with a comparable study by Doi et al. (2024) [52], who showed that plaque-control record (PCR) values derived from IOS images exceeded direct visual scores mainly on labial and palatal surfaces of maxillary anterior teeth, whereas the most considerable discrepancies occurred on posterior sites.

Several methodological factors can explain this phenomenon. Unlike most experimental protocols, our scans were acquired without plaque-disclosing dye, a step that improves chromatic contrast and has been shown to boost IOS sensitivity, especially on lingual and posterior surfaces [5, 52]. Image quality in the molar region is often reduced by optical shadowing and the restricted angulation of the scanner tip. This challenge has also been observed in studies comparing 2-D photographs with 3-D IOS images. It is particularly evident on the lingual surfaces of the lower jaw, where anatomical conditions frequently prevent the scanner from capturing an ideal image [6].

### Strengths and limitations

The study was conducted on a well-characterized longitudinal cohort, enabling the examination of a large population-based sample. The use of a standardized scanning protocol and calibrated, blinded examiners ensured high methodological rigor and supported the reproducibility of the results. A key limitation is the lack of a histological reference standard to validate the structural findings. The low disease burden strata limited high-severity case counts, mainly because of participants’ age, but also due to selection bias inherent to the study. A further limitation relates to the assessment of missing teeth. During chairside examinations, participants could be asked directly about the reasons for tooth loss (e.g., caries or MIH), thereby allowing background information to be incorporated into the interpretation of diagnostic findings. Such anamnesis-based clarification was not possible in the remote evaluation of scan data. As a result, it often remains unclear whether extractions were due to caries, MIH, or a combination of both – given that MIH-affected teeth are known to be more susceptible to carious decay [53, 54].

Moreover, the interpretation of agreement statistics in this study must be considered in the context of the low caries prevalence observed in the sample. The large majority of assessed tooth surfaces were clinically sound, which inevitably influences the magnitude of the weighted percent agreement ($\:{pa}_{w}$). However, this should not be interpreted as an artificial inflation of the results. The high $\:{pa}_{w}$ values reflect a genuine clinical finding: both the direct examination and the 3D scan reading independently and consistently identified the same surfaces as sound, with only rare discordant observations. The agreement observed is therefore substantively meaningful and not merely a consequence of a statistical ceiling effect.

At the same time, it is important to acknowledge that in low-prevalence settings $\:{pa}_{w}$ does not adjust for agreement attributable to chance alone. When one category predominates in a dataset, two examiners assigning scores may achieve high concordance even without strong methodological alignment. For this reason, $\:{pa}_{w}$ alone is not sufficient as a measure of agreement when disease prevalence is very low and should be interpreted alongside kappa. Conversely, Cohen’s weighted kappa (*κ*) is also sensitive to skewed prevalence distributions, a phenomenon often referred to as the prevalence paradox [37], which can lead to comparatively low kappa values despite high observed agreement. The divergence between high $\:{pa}_{w}$ and lower *κ* observed for initial caries lesions (ICDAS 1–2) in the present study reflects this statistical characteristic. Reporting both statistics together therefore provides a more transparent and balanced description of agreement in samples with a low burden of disease.

## Conclusion

In summary, weighted percent agreement indicated strong diagnostic agreement between 3D scan reading and direct clinical examination across oral health parameters. For some categories — notably initial caries lesions and early MIH manifestations — kappa values were low, reflecting the known statistical limitations of kappa under skewed distributions and poorer diagnostic agreement in less pronounced findings. The confirmed potential of remote IOS assessment supports its integration into large-scale epidemiological studies and preventive care models.

## Data Availability

The dataset presented in this article cannot be shared publicly because of ethical and legal restrictions. The LIFE Child study is a study collecting potentially sensitive information. Publishing data is not covered by the informed consent provided by the study participants. Furthermore, the data protection concept of LIFE requires all (external as well as internal) researchers interested in accessing data to sign a project agreement. Researchers interested in accessing data from the LIFE Child study may contact the study by writing to forschungsdaten@medizin.uni-leipzig.de.
